# Impact of Serum Ferritin on the Pathophysiology of Attention-Deficit/Hyperactivity Disorder: What Is the Evidence?

**DOI:** 10.7759/cureus.103196

**Published:** 2026-02-08

**Authors:** Tânia Araújo, Marina Rodrigues, Dina Campos

**Affiliations:** 1 General and Family Medicine, Unidade de Saúde Familiar Alcaides de Faria, Barcelona, PRT; 2 Family Medicine, Unidade de Saúde Familiar Vida+, Vila Verde, PRT; 3 Family Medicine, Unidade de Saúde Familiar Gualtar, Braga, PRT

**Keywords:** adhd, attention deficit hyperactivity disorder, attention deficit hyperactivity disorder (adhd), ferritin, iron deficiency, iron metabolism, neurodevelopment, pediatrics

## Abstract

Attention-Deficit/Hyperactivity Disorder (ADHD) is one of the most prevalent neurobehavioral disorders in childhood. Although its pathophysiology is not fully understood, growing interest has focused on potentially modifiable factors, including iron metabolism. Serum ferritin, a marker of peripheral iron status, has been widely investigated in relation to ADHD due to iron’s role in dopamine synthesis and neurodevelopment. This narrative evidence-based review aims to critically synthesize and interpret current clinical and mechanistic evidence regarding the association between serum ferritin levels and the pathophysiology of ADHD in the pediatric population.

A literature search was conducted using PubMed and reference lists of relevant articles to identify studies published in the last decade that evaluated serum ferritin in children with ADHD. The available evidence included a limited number of clinical trials, systematic reviews, and one meta-analysis, with heterogeneous methodologies and outcomes. While some studies reported lower serum ferritin levels in children with ADHD and associations with symptom severity, others did not confirm these findings.

Overall, current evidence is inconsistent and insufficient to support a definitive association between low serum ferritin levels and ADHD pathophysiology or to recommend routine ferritin assessment in clinical practice. Further well-designed, longitudinal studies are needed to clarify the role of iron status in ADHD and its potential clinical implications.

## Introduction and background

Attention-Deficit/Hyperactivity Disorder (ADHD) is one of the most prevalent neurobehavioral disorders in children worldwide, both in developed and developing countries. ADHD affects between 5 to 15% of school-aged children worldwide [1] and is more common in males [2]. According to the literature, discrepancies in prevalence may be explained by heterogeneity in diagnostic criteria, symptom assessment methodologies, and functional impairment [3].

One of the main characteristics of ADHD is excessive inattention inappropriate for the child’s age, as well as increased impulsivity and hyperactivity, with a negative impact [4]. According to the Diagnostic and Statistical Manual of Mental Disorders, fifth edition (DSM-5), children with ADHD may present predominantly inattentive, hyperactive/impulsive, or combined presentations. Regardless of subtype, quality of life is frequently affected, sometimes resulting in learning difficulties and impaired social functioning [5,6].

ADHD results from a combination of genetic and environmental factors [7]. Although it is considered a predominantly hereditary condition [8], environmental factors have increasingly been recognized as contributors to its etiology [9]. As some of these risk factors are potentially modifiable, several studies have focused on nutritional deficiencies, particularly iron deficiency [10], as well as other elements such as zinc.

Iron deficiency has been proposed as a potential risk factor in ADHD pathophysiology, with several observational studies reporting associations with cognitive, motor, social, and emotional functioning in children [6,11,12]. Iron acts as a cofactor for dopamine synthesis, a neurotransmitter involved in the modulation of several cognitive functions, and its deficiency has been associated with ADHD [13].

Given that serum ferritin levels are considered a reliable indicator of body iron stores, serum ferritin has been widely used in studies addressing this topic. Several studies suggest that children with ADHD have lower ferritin levels than healthy controls [14-16], while others report associations between lower ferritin levels and increased symptom severity [16]. This variability and inconsistency in findings has already been highlighted in earlier systematic reviews [17].

The objective of this review was to evaluate the impact of low serum ferritin levels on the pathophysiology of ADHD in the pediatric population.

## Review

Methods

Two independent researchers conducted a literature search in PubMed, the Cochrane Library, Evidence-Based Medicine Online, and the Portuguese Medical Repository (Repositório Médico Português - RMP). The search strategy used the following Medical Subject Headings (MeSH) terms: “Attention Deficit Disorder with Hyperactivity”, “Ferritins”, and “Pediatrics”, combined with the Boolean operator AND. Articles published within the last 10 years (between 2013 and 2023), written in English or Portuguese, were considered eligible. This timeframe was selected to ensure the inclusion of recent and methodologically robust evidence, reflecting advances in ADHD diagnostic criteria, iron metabolism assessment, and study design. The types of articles included were narrative reviews with a clear evidence-based methodology, systematic reviews, meta-analyses, randomized controlled trials, and clinical practice guidelines. Narrative reviews were included only when they explicitly described their literature search strategy and critical appraisal of the available evidence.

In addition, clinical practice guidelines were consulted in the following repositories: National Guidelines Clearinghouse, National Institute for Health and Care Excellence (NICE), and the Canadian Medical Association. No relevant guidelines addressing the study objective were identified.

The PICO model (Population, Intervention, Comparison, Outcome) was used as a guiding framework to structure the research question and define inclusion criteria for this narrative review (Table 1). 

**Table 1:** Inclusion criteria according to the PICO model ADHD: Attention-Deficit/Hyperactivity Disorder; PICO: Population, Intervention, Comparison, Outcome.

Population	Pediatric population with ADHD
Intervention	Serum ferritin measurement
Comparison	Healthy individuals
Outcome	Low ferritin levels in the pathophysiology of ADHD

This approach was chosen to ensure transparency and consistency in study selection, despite the narrative nature of the review. Study selection was performed independently by two researchers. Initially, titles were screened, followed by abstract review and full-text assessment. In cases of disagreement, a third reviewer evaluated the article.

The quality of evidence and strength of recommendations of the included studies were assessed using the Strength of Recommendation Taxonomy (SORT) of the American Academy of Family Physicians [16]. 

Results

The initial search identified 39 articles. Twelve articles were excluded due to duplication, including studies already incorporated into previous reviews. Ten articles were excluded after title screening, eight after abstract review, and four after full-text evaluation.

**Figure 1:**
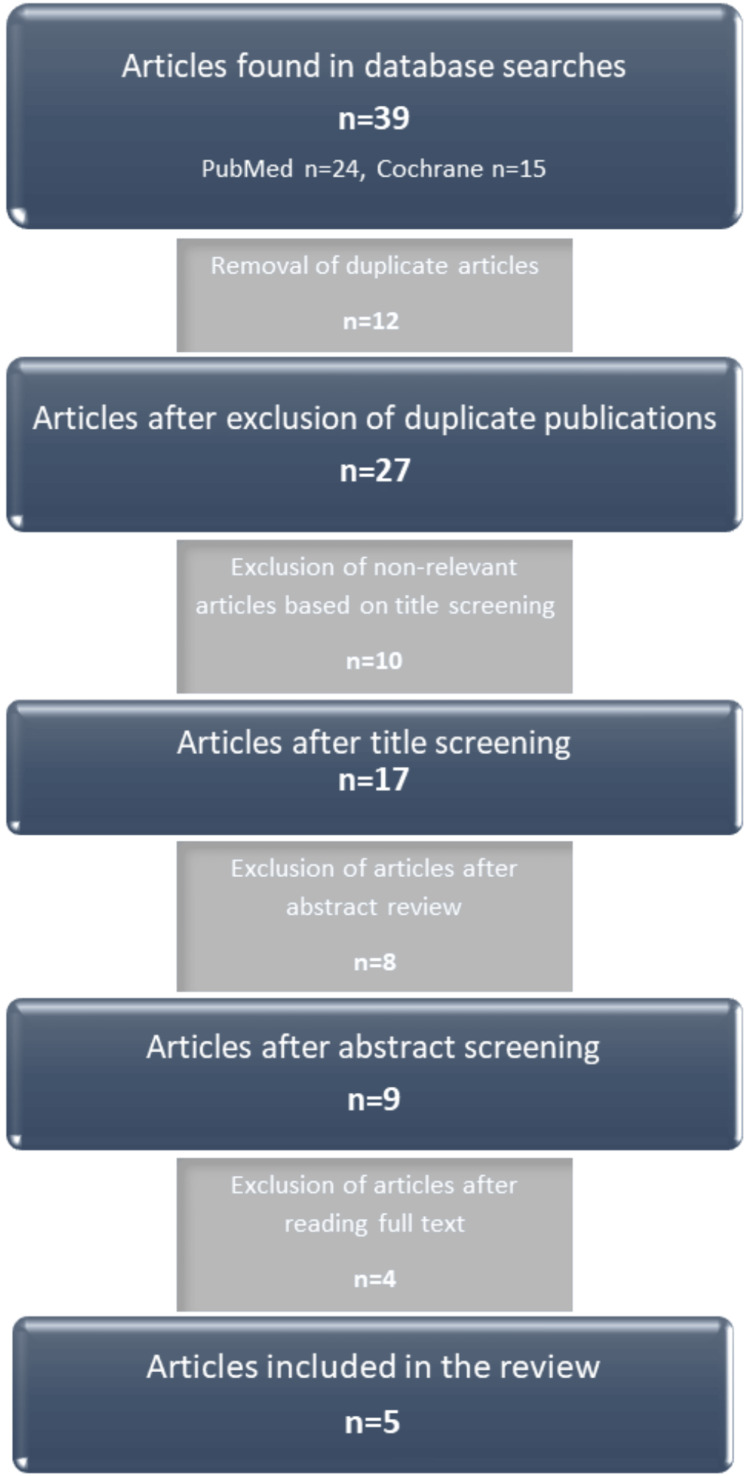
Flowchart of article selection

Ultimately, five articles met the inclusion criteria and were included in this review: one meta-analysis, one systematic review, and three clinical trials. All studies primarily aimed to compare iron concentrations, either ferritin alone or in combination with other iron parameters, between children with ADHD and healthy controls. The main characteristics of the included studies are summarized in Figure 1.

Meta-analysis

The included meta-analysis [18], published in 2018, evaluated peripheral iron levels and explored the association between iron deficiency, ADHD risk, and symptom severity. Serum ferritin levels were significantly lower in children with ADHD compared to controls (p=0.013), although substantial heterogeneity across studies was observed. This heterogeneity was mainly related to differences in study populations, ferritin cut-off values, age ranges, and methodological approaches across the included studies.

A subgroup analysis, including only unmedicated children, showed no significant differences in serum ferritin levels between groups (p=0.283). Age was identified as a moderating factor, with differences in ferritin levels increasing with age, possibly reflecting age-related variations in reference ranges within the pediatric population.

ADHD symptom severity was significantly higher in children with iron deficiency (p=0.002). A significant association between ADHD and iron deficiency was also observed (OR=1.636, p=0.031), although heterogeneity remained considerable.

Systematic review

A systematic review [19], published in 2021, analyzed differences in serum iron, ferritin, and brain iron concentrations in children with ADHD, with included studies covering a wide and variable age range. Twenty case-control studies were included, conducted predominantly in the Middle East, Asia, Europe, North America, and South America.

Serum ferritin results were highly variable, with mean values ranging from 12.9 ± 1.6 ng/mL to 134.2 ± 14.8 ng/mL in children with ADHD and from 12.7 ± 1.1 ng/mL to 110.3 ± 6.5 ng/mL in controls. Potential explanations for this variability included differences in medication history, lack of washout periods, limited control of confounding factors, and variability in ferritin measurement techniques. In addition, serum ferritin reference ranges are known to vary according to age and sex in pediatric populations, reflecting developmental changes in iron metabolism. Ferritin is also an acute-phase reactant and may be influenced by inflammatory status. In contrast, well-established race- or ethnicity-specific ferritin reference ranges in children are limited and not consistently defined, which may further contribute to variability across studies.

Only half of the included studies reported significantly lower ferritin levels in children with ADHD. Studies evaluating brain iron concentrations consistently reported lower levels in the ADHD group, particularly in the thalamus. However, the relationship between serum ferritin and brain iron remains unclear.

The review suggested that iron supplementation may reduce ADHD symptom severity, although the authors emphasized the need for further studies and highlighted uncertainty regarding whether supplementation could prevent or mitigate ADHD symptoms.

Clinical trials

A cross-sectional observational study, conducted in Egypt in 2018 [20], analyzed biochemical and neuroclinical characteristics in children with ADHD, epilepsy, both conditions, and healthy controls. Serum ferritin levels were significantly lower in children with ADHD compared to controls and other groups. However, the inclusion of children with epilepsy represents a potential confounding factor, as epilepsy and its treatment may independently affect iron metabolism.

Another cross-sectional study, conducted in Turkey in 2020 [21], evaluated ferritin levels, zinc status, and sensory processing in preschool-aged children with ADHD symptoms. No significant differences in ferritin levels were observed between groups, and no association between low ferritin and sensory deficits was identified.

A third cross-sectional study, published in 2022 [22], compared ferritin, vitamin B12, folic acid, and inflammatory markers in children with ADHD, autism spectrum disorder, and healthy controls. Ferritin levels did not differ significantly between groups, although a non-significant correlation with behavioral disturbances was observed. However, the inclusion of children with autism spectrum disorder represents a potential confounding factor, which may limit the direct comparability of these findings with studies focusing exclusively on ADHD populations.

Table 2 provides a comprehensive summary of the studies included in this narrative review, outlining their main characteristics, study designs, populations, and key findings regarding the association between serum ferritin levels and ADHD.

**Table 2:** Summary of articles ADHD: Attention-Deficit/Hyperactivity Disorder; DSM: Diagnostic and Statistical Manual of Mental Disorders; ASD: Autism Spectrum Disorder; SR: Strength of recommendation; LE: Level of evidence.

Reference	Methodology	Results	Limitations	SR/LE
Tseng et al. (2018) [18]	Meta-analysis; 17 articles included (13 with ferritin assessment) -ADHD (N=1560; mean age 10.1 ); Controls (N=4691; mean age 10.4); ADHD diagnostic criteria: mostly DSM IV	Peripheral ferritin was significantly lower in children with ADHD - High heterogeneity of results; ADHD severity was significantly higher in children with iron deficiency - No evidence of heterogeneity; Statistically significant association between ADHD and iron deficiency (OR=1.636; p=0.031) - Significant heterogeneity	Small number of articles included Possible confounders: iron intake in diet, different methods of iron parameter measurement, limited cohort studies with long-term follow-up	SR B LE 2
Degremont et al. (2021) [19]	Systematic review; 20 case-control studies N= 5191 (ADHD 2209; healthy controls 2982); Mean age 4 to 12.6 years; Diagnostic criteria: mostly DSM IV/V; Only two studies included medicated children; Children supplemented with iron were excluded.	Only 10 of 18 studies evaluating serum ferritin reported significantly lower levels in children with ADHD compared to controls, with high variability and inconsistent results. Brain iron concentrations were consistently lower in the ADHD group, particularly in the thalamus.	Compromised cause-effect relationship due to study design; Possible confounders: dietary iron intake, intake of nutrients that enhance iron absorption (vitamin C), oxidative and psychological stress, medication, inflammatory states	SR C LE 3
Topal et al., (2021) [22]	Cross-sectional observational study; N=203 (ADHD 61; ASD 72; healthy controls 70); Mean age 8.5 years (range 2-17 years); ADHD diagnostic criteria: Ages 6-18 years: Schedule for Affective Disorders and Schizophrenia for School-Age Children <6 years: DSM-V; Excluded children with chronic comorbid medical conditions, vitamin supplements, chronic medication, infectious disease at the time of assessment	No significant differences between groups (ADHD: 25.17±13.34; controls: 20.99±13.86) Ferritin levels tended to correlate with behavioral problems (rho=-0.51, p<0.01), but statistical significance was not reached	Dynamic changes in nutritional status over time; Potential comorbidities in children with ADHD/ASD were not controlled for	LE 2
Yoldaş et al. (2020) [21]	Cross-sectional observational study; N=44 (ADHD 22; healthy controls 22); Preschool age: 48-72 months; Developmental assessment: Denver II- Developmental Screening Test	The majority of children in the study group (n=14, 63.6%) and the control group (n=13, 59.1%) had normal ferritin levels. No statistically significant difference (p=0.757)	Sample size; Preschool age; age-related variability in ferritin levels may affect comparability.	LE 2
Abd El Naby et al. (2018) [20]	Cross-sectional observational study; N=100 (25 children/group): I – Control; II – ADHD; III – Epilepsy; IV - ADHD and epilepsy Diagnostic criteria: DSM-IV; Excluded children with chronic systemic diseases or other neurological or psychological disorders, uncontrolled epilepsy, IQ <70, or under nutritional supplements; Age range not reported/not specified by authors	Significantly lower serum ferritin in the ADHD group (110.27±6.64 ng/ml) compared to: controls (134.23±14.82 ng/ml); epilepsy (159.66±33.17 ng/ml); ADHD with epilepsy (203.04±50.64 ng/ml)	Sample size	LE 3

The table allows for a structured comparison of the available evidence and facilitates a clearer understanding of the existing literature on this topic.

Across the included studies, reporting of key potential confounders, such as dietary intake, pharmacological treatment, and comorbid conditions, was inconsistent, limiting their systematic evaluation in the results synthesis.

Discussion

This study allowed for a comparison of serum ferritin concentrations in children with ADHD and healthy children, highlighting inconsistencies in the findings of different studies. Lower serum ferritin levels in children with ADHD have been reported in the literature [16]. A systematic review emphasized the inconsistency of results across studies [19]. Other authors identified lower ferritin levels in children with ADHD, whereas some studies did not observe this association [20-22]. The causal role of iron deficiency in ADHD remains controversial [23].

Serum ferritin is an early marker of iron deficiency, as it is sensitive to fluctuations in iron levels. Additionally, it also serves as a marker of inflammation, infection, and oxidative stress [26]. Therefore, variations in ferritin levels are expected under different conditions, which may explain the heterogeneity of the reported results. Across the different studies included in this review, the technique used to measure serum ferritin was not always specified. As a result, the reported values may not be directly comparable, which could also contribute to the high heterogeneity of the results. Differences in patient populations across studies, including wide age ranges (preschool vs. school-aged children) and the inclusion of comorbid conditions such as epilepsy or autism spectrum disorder, may further contribute to the observed heterogeneity of findings. Therefore, this factor represents a limitation of the study. In addition, serum ferritin reference ranges are known to vary according to age and sex in pediatric populations, reflecting developmental changes in iron metabolism. Although most studies applied age-appropriate pediatric reference ranges, variability in age distribution across study populations may still contribute to heterogeneity and limit direct comparability of results.

To further clarify these heterogeneous results, Tseng et al. [18] analyzed a subgroup of unmedicated children (included in three of the 17 studies reviewed) and found no significant differences between the ADHD and control groups. It is important to highlight that most of the included studies did not provide information on prescribed medication. Pharmacological treatment for ADHD is primarily based on the use of psychostimulants, with methylphenidate being the most used drug. Some side effects are associated with its use, particularly appetite suppression, which may lead to reduced food intake and, consequently, lower iron consumption [24]. In the systematic review by Degremont et al. [19], only unmedicated children at the time of the study were included. However, these children might have been previously medicated, with no mention of a washout period, meaning they could still have been under the influence of the drugs. Therefore, the lack of clarity regarding prior treatments across different studies may represent a limitation, as medication use could impact serum ferritin.

Taken together, the available literature suggests a potential association between low serum ferritin levels and ADHD. However, this relationship remains inconsistent and highly context-dependent. Differences in study design, age groups, ferritin cut-off values, assessment of inflammatory status, and control of relevant confounders substantially limit comparability across studies. Importantly, most available data derive from observational designs, precluding causal inference and raising the possibility that low ferritin may act as a marker of broader nutritional, inflammatory, or socioeconomic factors rather than a direct contributor to ADHD pathophysiology. Consequently, current evidence supports a cautious interpretation of ferritin alterations in ADHD, emphasizing the need for integrated clinical and biological frameworks when evaluating iron status in affected children.

Tseng et al. [18] concluded that children with serum iron deficiency were more likely to have ADHD and exhibited greater symptom severity. Another study conducted by Kwon et al. found that children who received iron supplementation showed a reduction in ADHD symptoms [25]. Thus, iron deficiency may be associated with ADHD symptom severity. Topal et al. [22] reported similar findings, although without statistical significance, correlating ferritin levels with behavioral disorders.

Importantly, none of the included studies demonstrated a clear dose-response relationship between serum ferritin levels and ADHD symptom severity or clinical improvement, further limiting the definition of clinically meaningful ferritin thresholds or prediction of treatment response.

From a mechanistic perspective, exploring the relationship between iron status and dopaminergic function may help contextualize the inconsistent clinical findings observed across studies and provide a biological rationale for further investigation. Dopamine is a neurotransmitter that plays a fundamental role in modulating various cognitive functions, including psychomotor activity and executive functions. Alterations in dopaminergic signaling have been described as playing a central role in the pathophysiology of ADHD [26]. In the brain, iron acts as a cofactor for the enzyme tyrosine hydroxylase, which is essential for dopamine synthesis [9]. Therefore, iron deficiency may negatively influence the production of this neurotransmitter [17], potentially impacting behavior. Degremont et al. [19] reported significantly lower brain iron concentrations in the ADHD group, particularly in the thalamus. The thalamus is responsible for modulating behavioral responses to environmental stimuli, and dopamine deficiency in this structure has been implicated in the pathophysiology of ADHD [13]. Given the impact of nutritional deficiencies in early life on brain development [27], Degremont et al. [19] suggested that iron deficiency during this critical period may negatively affect neuronal and behavioral development. However, Yoldaş et al. [21] did not find an association between ferritin levels and ADHD, despite studying a younger population of preschool-aged children, in contrast to most other studies, which focused on school-aged children.

Across the different studies, children receiving iron supplementation were excluded. However, the absence of dietary records prevented an accurate assessment of nutritional status and the quality of iron intake. Chen et al. [28] found that children with ADHD had a higher intake of iron and vitamin C, which enhances iron absorption, compared to the control group, resulting in higher serum iron levels. Therefore, future studies should consider characterizing iron and vitamin C intake, given the potential impact of these factors on the reported results.

The clinical trials included in this review have a small sample size, resulting in limited robustness [20-22]. Moreover, as case-control studies, they do not allow for the establishment of a cause-effect relationship. In addition, variability in participant characteristics - including age ranges, sex distribution, comorbid conditions, medication exposure, and potential ethnic or socioeconomic differences - as well as differences in study design and ferritin cut-off values, likely contributed to the heterogeneity of results across studies.

At present, there is no consensus regarding serum ferritin thresholds or treatment duration to guide iron supplementation in children with ADHD, and routine supplementation cannot be recommended without confirmed iron deficiency.

In summary, future longitudinal studies with large patient samples are needed, incorporating controls for potential confounders, such as pharmacological treatment and dietary patterns. This approach would help clarify the conflicting results observed across different studies and minimize the influence of confounding factors.

## Conclusions

Based on the available evidence, this review suggests that current data are insufficient to support a definitive association between lower serum ferritin levels and ADHD in children, given the inconsistency of findings across studies (strength of recommendation: B). Although some studies report lower ferritin levels or greater symptom severity in the context of iron deficiency, results remain heterogeneous and methodologically limited. Further well-designed, longitudinal studies with larger samples and adequate control of confounding factors are needed to clarify the role of serum ferritin in the pathophysiology of ADHD.

In addition, independent of ADHD diagnosis, adequate iron intake during infancy and early childhood remains essential for optimal neurodevelopment. Given the well-established cognitive and developmental consequences of iron deficiency, promoting appropriate nutrition and early identification of iron deficiency should be considered a cornerstone of pediatric preventive care. Based on current evidence, routine ferritin screening in children with ADHD cannot be recommended in the absence of documented or suspected iron deficiency.
